# Formulation and Characterization of Soybean Oil-in-Water Emulsions Stabilized Using Gelatinized Starch Dispersions from Plant Sources

**DOI:** 10.3390/molecules29091923

**Published:** 2024-04-23

**Authors:** Ankita Singh, Takumi Umeda, Isao Kobayashi

**Affiliations:** 1 Institute of Food Research, National Agriculture and Food Research Organization, 2-1-12 Kannodai, Tsukuba 305-8642, Ibaraki, Japan; s2230319@s.tsukuba.ac.jp (A.S.); umedat307@affrc.go.jp (T.U.); 2 Graduate School of Science and Technology, University of Tsukuba, 1-1-1 Tennodai, Tsukuba 305-8572, Ibaraki, Japan; 3 School of Integrative and Global Majors, University of Tsukuba, 1-1-1 Tennodai, Tsukuba 305-8577, Ibaraki, Japan

**Keywords:** starch, gelatinization, clean label, natural, physical modification, emulsification, homogenization

## Abstract

Consumers are concerned about employing green processing technologies and natural ingredients in different manufacturing sectors to achieve a “clean label” standard for products and minimize the hazardous impact of chemical ingredients on human health and the environment. In this study, we investigated the effects of gelatinized starch dispersions (GSDs) prepared from six plant sources (*indica* and *japonica* rice, wheat, corn, potatoes, and sweet potatoes) on the formulation and stability of oil-in-water (O/W) emulsions. The effect of gelatinization temperature and time conditions of 85–90 °C for 20 min on the interfacial tension of the two phases was observed. Emulsification was performed using a primary homogenization condition of 10,000 rpm for 5 min, followed by high-pressure homogenization at 100 MPa for five cycles. The effects of higher oil weight fractions (15–25% *w*/*w*) and storage stability at different temperatures for four weeks were also evaluated. The interfacial tension of all starch GSDs with soybean oil decreased compared with the interfacial tension between soybean oil and water as a control. The largest interfacial tension reduction was observed for the GSD from *indica* rice. Microstructural analysis indicated that the GSDs stabilized the O/W emulsion by coating oil droplets. Emulsions formulated using a GSD from *indica* rice were stable during four weeks of storage with a volume mean diameter (*d*_4,3_) of ~1 µm, minimal viscosity change, and a negative ζ-potential.

## 1. Introduction

An emulsion is formed by blending two or more liquids that do not naturally mix with one liquid dispersed as spherical droplets within the other phase using emulsification devices. In addition to basic emulsions, such as oil-in-water (O/W) or water-in-oil (W/O), multiple emulsions are produced by incorporating simple emulsions as the dispersed phase. Examples include double emulsions such as water-in-oil-in-water (W/O/W) and oil-in-water-in-oil (O/W/O) emulsions. It is imperative to maintain the stability of emulsions throughout their processing, storage, transportation, and usage for a specified duration. However, emulsions are prone to instability over time under varying temperatures and conditions, resulting in phenomena such as creaming, sedimentation, flocculation, coalescence, and emulsion phase inversion [1]. In general, emulsions are thermodynamically unstable; therefore, emulsion microstructures are kinetically stabilized using surfactants. Within the food industry, surfactants refer to any components utilized to improve the stability of an emulsion. They are categorized as emulsifiers or texture modifiers based on their mechanisms of action. The most commonly used emulsifiers are synthetic surfactants, which include small-molecule surfactants that are further classified as ionic, non-ionic, or zwitterionic [2]. Non-ionic surfactants have found widespread application in emulsion formation owing to their minimal toxicity, non-irritating nature, and ability to readily create emulsions through both high-energy and low-energy methods. Examples include glycerin fatty acid esters (including monoglycerides, diglycerides, and triglycerides), sugar ester surfactants (including sorbitan monooleate and sucrose monopalmitate), polyoxyethylene ether (POE) surfactants, and ethoxylated sorbitan esters (including Tweens and Spans) [3]. Zwitterionic surfactants are characterized by the presence of two or more ionizable groups with opposing charges in a single molecule. Common examples are phospholipids with “generally recognized as safe” status, such as lecithin. However, several natural phospholipids may not exhibit strong capabilities for emulsion formulation or stabilization when employed independently. Nevertheless, they may be effective when paired with cosurfactants. Another category is natural emulsifiers, which are further classified into biosurfactants and include saponins, glycolipids, and lipopeptides and bioemulsifiers, which are surface-active high molecular weight compounds sourced from microbes, such as hydrophobins, mannoproteins, and emulsan [4]. The most “label-friendly”, natural emulsifiers are proteins and polysaccharides. Caseins, whey proteins, egg proteins, legume proteins, and gelatin are food proteins that contain a variety of polar and nonpolar groups, along with anionic, neutral, and cationic amino acids distributed throughout their polypeptide chains. These features determine their electrostatic properties, affect their solubility in water, and govern their surface activity, thus influencing their role in stabilizing emulsions [5]. Commercially used natural emulsifier polysaccharides include gum arabic, pectin (from fruits such as apples and oranges), modified starch, and modified cellulose. They are mainly used as texture modifiers and weighing agents that increase the viscosity of the system, retard droplet movement, prevent gravitational separation, and prevent creaming or sedimentation [2]. Nevertheless, most commercially utilized polysaccharides are not entirely natural, because they undergo chemical modification by attaching nonpolar chains to starch or cellulose molecules. Therefore, extensive studies are required to explore the emulsification ability of natural polysaccharide sources to ensure “clean labels” for products.

Starch is the predominant carbohydrate in plants and is inexpensive and extremely diverse in size, shape, polysaccharides, and mineral composition based on its botanical origin. In addition to its use in food processing, starch has extensive applications in various sectors, including textiles, pharmaceuticals, the paper industry, adhesives, and bioplastics. The principal starch polysaccharides, amylose and amylopectin, primarily influence the physical characteristics of starches. Studies using X-ray diffraction on starch granules indicate their semi-crystalline nature, comprising approximately 30% of the granule mass as a crystalline region and 70% as an amorphous region [6]. Amylose comprises long, linear α-glucan molecules containing approximately 99% of (1-4)-α- and (1-6)-α- linkages. Conversely, amylopectin exhibits a heavily branched structure, with 95% (1-4)-α- and 5% (1-6)-α- linkages. Upon hydrothermal treatment, amylopectin aids in the water absorption, swelling, and pasting of starch granules, while amylose is more likely to inhibit these processes [7]. Starch applications depend on its properties, including solubility, viscosity, and hydrophobicity, which are determined by factors such as structure, polysaccharide ratio, degree of chain branching, degree of substitution, and molecular weight [8,9]. Different physical, chemical, and enzymatic starch modification methods have been employed to address the challenges associated with native starches, such as low hydrophobicity and solubility. Therefore, modification methods can improve the functional properties, that is solubility and hydrophobicity, required for starch applications [10].

Most encapsulation techniques employed for the preparation of starch matrices rely on methods such as spray drying, jet cooking, and emulsion crosslinking. In previous studies, using steam jet cooking at high temperature and steam, a two-phase mixture of starch formed a stable phase, and phase separation did not occur, even after prolonged standing or drying [11]. However, these approaches have various drawbacks, including environmental pollution, high energy expenditure, and safety considerations [12]. The chemical or enzymatic modification of starches presents disadvantages, including high cost and environmental insensitivity, and the fact that they are not truly “natural” emulsifiers may not satisfactorily meet clean label parameters. Food O/W emulsions stabilized by gelatinized starches are uncommon, despite the fact that starch is the most abundant natural polymer with emulsifying properties. In previous studies, oil-in-water (O/W) emulsions were stabilized using gelatinized native kudzu root, supernatant fractions of centrifuged GSDs, and mango kernel starches, without a commercial or synthetic emulsifier [6,13,14]. The limitations of previous studies can be summarized as a lack of information regarding the duration of storage stability under different temperature conditions, a narrow range of applications, and cost efficiency.

Therefore, the primary objective of this study was investigating the formulation characteristics of GSDs stabilized oil-in-water emulsions and their storage stability, along with exploring the mechanisms behind emulsion stabilization by GSDs. Physical modification of starch was carried out using gelatinization, which can be defined as the hydrothermal disordering of crystalline structures in native starch granules that causes loss of crystallinity, leaching of amylose, increase in viscosity, and swelling of the granules. Physical modification by gelatinization is entirely a natural starch modification process that does not require any chemical group’s introduction to starches, therefore, it aligns with clean label requirements. The properties of the starch-water system were further improved on mechanical dispersion by a combination of homogenization techniques. A high-pressure homogenizer was selected to prepare the emulsions, considering the physicochemical properties of the starches and the set objectives. The selection of the starch sources was based on their ample starch content, year-round worldwide accessibility, and cost efficiency. The reason for selection of soybean oil is that it has been chosen in previous research as a model vegetable oil for use as the dispersed phase in emulsification. GSDs were prepared using starches from cereals (*indica* and *japonica* rice, wheat, and corn) and vegetable sources (potatoes and sweet potatoes), and the effects of gelatinization and high-pressure homogenization on the formulation mechanism of O/W emulsions and their stability during storage were investigated.

## 2. Results and Discussion

### 2.1. Effect of GSDs on the Formulation and Storage Stability of O/W Emulsions

After gelatinization, the calculated volume mean diameter (d_4,3_) of *indica* rice (R), wheat (W), corn (C), potato (P), sweet potato (SP), and *japonica* rice (JRS) GSDs, in descending order of d_4,3_, were PGSD (135.03 ± 2.89 µm), WGSD (47.66 ± 0.05 µm), SPGSD (42.10 ± 1.68 µm), CGSD (40.31 ± 0.22 µm), RGSD (13.67 ± 0.16 µm), and JRSGSD (10.61 ± 0.05 µm). An increase in d_4,3_ to form GSDs was observed for all the starch powders (Figure S1). Calculated d_4,3_ was largest for potato starch (PS; 49.42 ± 0.71 µm), sweet potato starch (SPS; 16.90 ± 0.19 µm), wheat starch (WS; 16.15 ± 0.06 µm), corn starch (CS; 13.16 ± 1.65 µm), and *indica* rice starch (RS; 13.12 ± 0.65 µm), and smallest for *japonica* rice starch (JRS; 5.61 ± 0.76 µm). Increases in d_4,3_ were attributed to starch granule swelling [6] as evident from the GSD optical micrographs (Figure S2). Storage time had almost no impact on the d_4,3_ values of the GSDs. The smaller particle size (d_4,3_) of *indica* and *japonica* rice GSDs was a desirable stability factor because emulsions formulated using them as the continuous phase would have smaller droplets. This observation was consistent with the findings of prior research [15] which reported that the small particle sizes of the continuous-phase constituents play an important role in the adsorption capacity of the dispersed phase.

**Figure 1:**
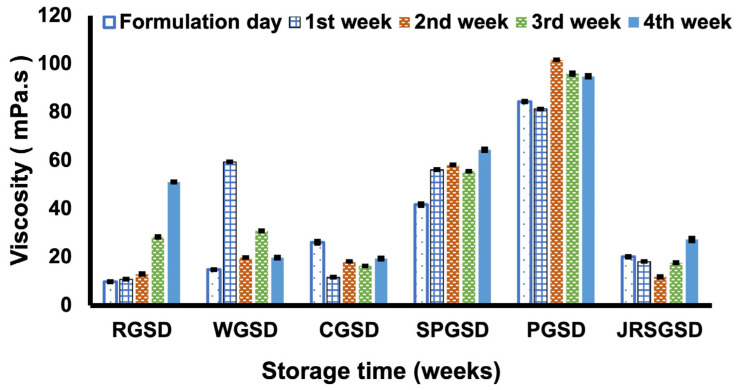
Viscosity evaluation during four weeks of storage for *indica* rice gelatinized starch dispersion (RGSD), wheat gelatinized starch dispersion (WGSD), corn gelatinized starch dispersion (CGSD), sweet potato gelatinized starch dispersion (SPGSD), potato gelatinized starch dispersion (PGSD), and *japonica* rice gelatinized starch dispersion (JRSGSD).

The viscosities of all GSDs decreased with increasing shear rate (Figure S3). During the four weeks of storage, all GSDs showed an increasing trend in viscosity, although the rate of increase was small (Figure 1). The apparent increase in viscosity could be a consequence of the increased molecular interactions between the polysaccharides and water, which creates a gel network [16]. Based on the calculated proportions of polysaccharides (Table S1), *indica* rice, wheat, and corn had low amylopectin content, whereas potato, sweet potato, and *japonica* rice had high amylopectin content. Higher amylopectin content increases the viscosity, pasting, and gelling characteristics of starch–water systems. However, PGSD (94.40 mPa·s) and SPGSD (63.74 mPa·s) had higher viscosities due to their large d_4,3_ and higher amylopectin content. Consequently, emulsions formulated using these starches had faster retrogradation and creaming. In contrast, despite having a higher amylopectin content, the small d_4,3_ of JRGSD led to a suitable range of viscosities needed for a stable emulsion formulation. Apart from the polysaccharide proportions and granule size, several other factors associated with starch affect its physicochemical properties, such as shape (Figure S4), molecular weight, degrees of branching and substitution, and the presence of trace minerals. According to previous reports [17,18,19], the intrinsic viscosities of amylopectin were higher than those of amylose and the intrinsic viscosity increases with molecular weight. Our findings are in accordance with previous reports as the viscosity of RGSD, JRGSD, WGSD, and CGSD were in the smaller range and SPGSD and PGSD were in the higher range (Figure 1). So, it can be estimated that sweet potato and potato were high molecular weight starches and *indica* rice, *japonica* rice, wheat, and corn starch were low molecular weight starches. The moderate viscosity range exhibited by GSDs (RGSD, WGSD, CGSD, and JRGSD) contributes to emulsion stability by slowing the movement of oil droplets, which limits flocculation and coalescence phenomena [20].

**Figure 2:**
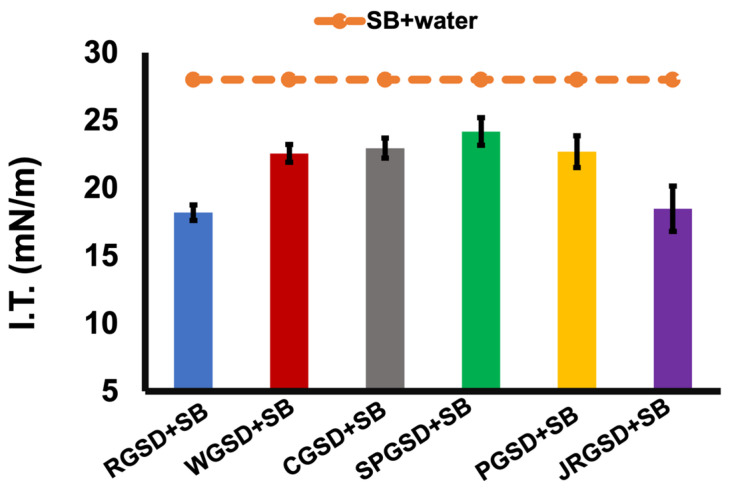
Interfacial tension of starch (R, *indica* rice; W, wheat; C, corn; SP, sweet potato; P, potato; JR, *japonica* rice) gelatinized starch dispersions (GSDs) with soybean oil (SB). Control: Milli-Q water and SB.

The reduction in interfacial tension is considered the primary mechanism for the formation of O/W emulsions [1]. All the GSDs (3% *w*/*w*) were found to reduce the interfacial tension (γ) with soybean oil (Figure 2). The interfacial tension (γ) between Milli-Q water and soybean oil was 28.56 mN/m at 25 °C. Among all the GSDs, the highest interfacial tension reduction was observed with *indica* rice starch dispersion (18.19 mN/m), followed by gelatinized *japonica* rice starch dispersion (18.48 mN/m), at 25 °C. A gelatinization temperature of 90 °C and time of 20 min were selected from a previous study [6]. According to this study, the decrease in γ was directly correlated with gelatinization time and reached a constant value after 20 min of heating at 90 °C. The GSDs were also homogenized at 10,000 rpm for 5 min before emulsification to further reduce their d_4,3_, which would improve their interfacial tension-lowering capacity owing to particle size reduction. The gelatinization temperature enhances starch solubility in water and induces melting of crystalline regions, forming a polymer network. The γ-reduction could be attributed to the formation of helical inclusion complexes with the oil phase during high shear and high-pressure homogenization. These complexes may reside at the newly formed oil-water phase interface. The adsorption of the GSDs on the dispersed-phase droplets was confirmed by optical microscopy. Furthermore, the molecular weight of starch polysaccharides considerably influences the diffusion kinetics at the interface [17], thereby influencing the interfacial tension.

### 2.2. Formulation and Characterization of the O/W Emulsions Stabilized by GSDs

**Figure 3:**
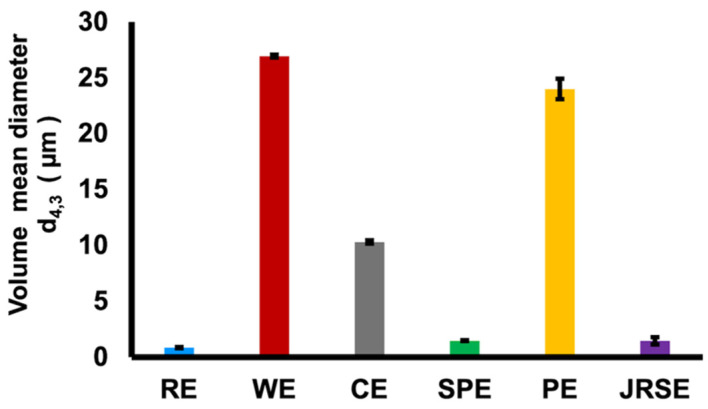
Volume mean diameter (d_4,3_) and Zeta potential of the GSD stabilized soybean oil-in-water (O/W) emulsions. RE: *indica* rice GSD stabilized (O/W) emulsion, WE: Wheat GSD stabilized (O/W) emulsion, CE: Corn GSD stabilized (O/W) emulsion, SPE: Sweet potato GSD stabilized (O/W) emulsion, PE: Potato GSD stabilized (O/W) emulsion, JRSE: *japonica* rice GSD stabilized (O/W) emulsion.

After formulation, all GSD-stabilized O/W emulsions were analyzed for particle size distribution and d_4,3_ (Figure 3). We observed that *indica* rice GSD-stabilized O/W emulsion (RE) had the smallest d_4,3_ of 0.87 ± 0.06 μm, followed by *japonica* rice GSD-stabilized O/W emulsion (JRSE; d_4,3_: 1.47 ± 0.33 μm), sweet potato GSD-stabilized O/W emulsion (SPE; d_4,3_: 1.49 ± 0.02 μm), and corn GSD-stabilized O/W emulsion (CE; d_4,3_: 10.32 ± 0.16 μm). In contrast, the largest particle size was observed in wheat GSD-stabilized O/W emulsion (WE; d_4,3_: 26.95 ± 0.13 μm) and potato GSD-stabilized O/W emulsion (PE; d_4,3_: 24.01 ± 0.9 μm). The observed particle size seems to be directly related to the d_4,3_ of the corresponding GSDs used in emulsification. The viscosity of the formulated emulsions (Figure S5) decreased with an increase in the shear rate, suggesting the non-Newtonian character of the GSD-stabilized emulsions. The viscosities of PE and WE were the highest after formulation, whereas those for CE and SPE were the lowest. This is possibly due to the higher d_4,3_ and molecular weight of PE and larger d_4,3_ of WE as well as the interactions of polysaccharides with the oil phase. The optimum viscosity range observed in the RE and JRSE emulsions is desired to achieve storage stability. The GSD-stabilized O/W emulsions were observed to carry small negative charges (25–40 mV) (Table S2) which create weak steric repulsive charges that play an important role in emulsion stabilization. The largest surface zeta potential (ζ-potential) was observed with JRSE, followed by RE, whereas the lowest ζ-potential was observed in WE, followed by PE. Thermal studies of starches validated the zeta potential observations, as they have shown that higher amylopectin content starches increase the enthalpy of gelatinization (ΔH_gel_), hence, there is a smaller degree of starch granule modification, and consequently fewer ionic groups will be available to exert ζ-potential, therefore emulsification efficiency is reduced [17,21]. Contrary to this, JRSE, despite having high amylopectin content, possesses a high ζ-potential, indicating that its smaller d_4,3_ (1.47 μm), the lesser viscosity of JRGSD, and the low molecular weight of *japonica* rice starch could have resulted in its high electronegative property. Zeta potential serves as an indicator of the surface charge of the particles. Native starches typically exhibit low negative charges because of the presence of polar hydroxyl and carbonyl groups on their molecular chains. However, during modification through gelatinization, more of these hydroxyl and carbonyl groups are exposed because of the breakdown of the crystalline structures and molecular interactions with water. Subsequently, they undergo deprotonation or ionization [20]. Therefore, it is evident that the size, crystallinity, and molecular weight of GSDs affect the electrical charge of the corresponding emulsions.

Theoretical support for the mechanism of polysaccharides as emulsifying agents was provided by the self-consistent field calculations conducted in a previous study [8]. These calculations suggest that amylopectin forms a more compact layer at the interface, whereas amylose forms less dense but more extended films, leading to strong, longer-range steric repulsion. A higher or lower content of either polysaccharide leads to emulsion instability by accelerating retrogradation rates during storage. Emulsion characteristics are dependent on thermal properties of all the native starch powders, namely onset temperature (T_O_), peak temperature (T_P_), conclusion temperature (T_C_), and gelatinization enthalpy (ΔH_gel_) (Table S3). We observed higher ΔH_gel_ (~15–20 J/g) for starches with higher amylopectin content (SPS, PS, and JRS), which agrees with a previous report [21]. In contrast, low ΔH_gel_ (~10–13 J/g) was observed in starches with low amylopectin content (RS, WS, and CS).

**Figure 4:**
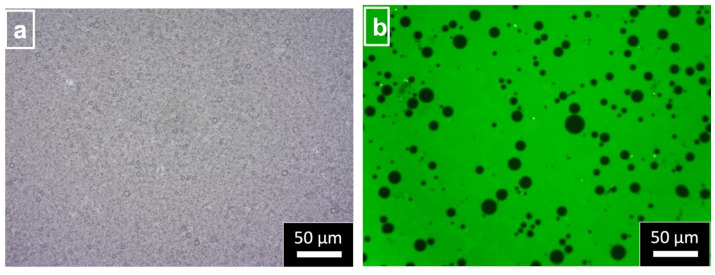
(**a**) Optical and (**b**) fluorescent micrographs of *indica* rice GSD stabilized O/W emulsion (RE, d_4,3_ 0.85 µm).

From optical micrograph and microstructural observations using fluorescent micrographs (Figure 4a,b), the formulation mechanism of the GSD-stabilized O/W emulsion can be estimated. The GSDs were observed to be coating and layering the dispersed phase oil droplets, and there were free GSDs maintaining the viscosity of the emulsion. This suggests that starch was successfully modified by gelatinization, and its amphiphilicity increased. The adsorption and layering of GSDs on dispersed-phase droplets were observed, suggesting their improved hydrophobicity. In contrast, the free GSDs present in the continuous phase are more hydrophilic, thereby increasing the viscosity of the emulsion system and preventing the movement of the oil phase and, hence, its corresponding coalescence. Smaller d_4,3_ of the GSDs was the most important characteristic, as we observed that RSE and JRSE formed the most stable emulsions, followed by SPE and CE. Their ΔH_gel_ was in the lower range, indicating a higher degree of gelatinization of their GSDs; consequently, more ionic groups are available to maintain the ζ-potential, thereby stabilizing these O/W emulsions. In contrast, PE and WE, due to the large d_4,3_ of their GSDs, had higher ΔH_gel_ so a lower degree of gelatinization; consequently, fewer ionic groups are available to maintain the ζ-potential, and as a result they form less stable emulsions.

### 2.3. Evaluation of Emulsion Stability during Storage at Three Different Temperatures

The emulsions formulated using 3% (*w*/*w*) GSDs from the six starches and 10% (*w*/*w*) soybean oil were stored at 5 °C, 25 °C, and 45 °C for four weeks to evaluate their stability in terms of change in d_4,3_, viscosity, ζ-potential, and microscopic and visual appearance.

For emulsions stored at low temperature (5 °C) for four weeks, all emulsions exhibited stable visual appearance, with no creaming. The ζ-potential was maintained during storage, thereby stabilizing the emulsions (Figure S6). The viscosity change was the lowest for RE, JRSE, and PE, whereas it increased exponentially for the remaining emulsions (Figure S7). The d_4,3_ values for RE were the smallest during storage, whereas d_4,3_ of WE, SPE, and JRSE increased for three weeks and then decreased in the fourth week (Figure S8). Although creaming was not observed in any emulsion, PE underwent phase separation as it oiled off. According to a previous study [22], proteins can form complexes with starch that inhibit retrogradation during refrigerated storage. Therefore, traces of protein present in starch GSDs might have a role in emulsion stability at 5 °C storage. Optical micrographs (Figure S9) showed that the dispersed-phase droplets were well coated with GSDs, and the emulsion droplets were uniformly distributed even after four weeks of storage.

**Figure 5:**
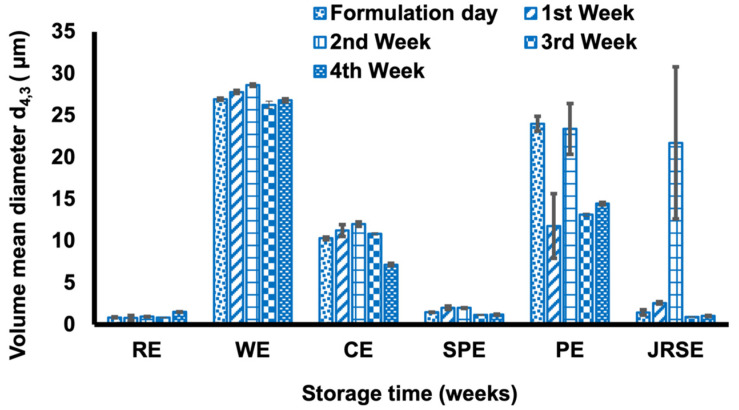
Volume mean diameter (d_4,3_) of the GSD-stabilized soybean O/W emulsions during four weeks of storage at 25 °C.

**Figure 6:**
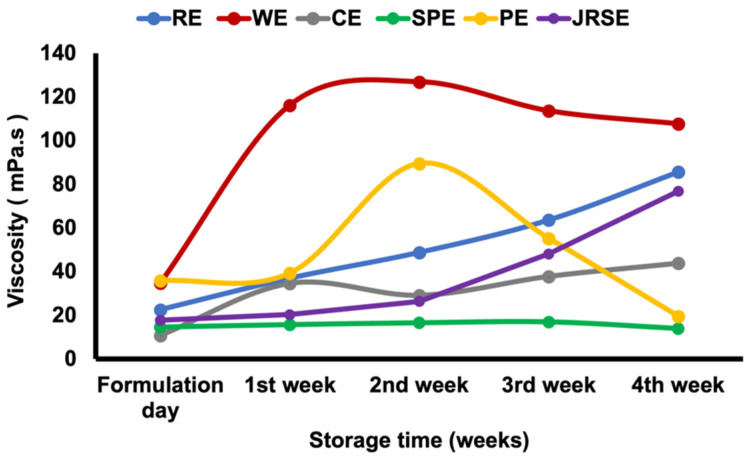
Viscosity of the GSD-stabilized soybean O/W emulsions during four weeks of storage at 25 °C.

**Figure 7:**
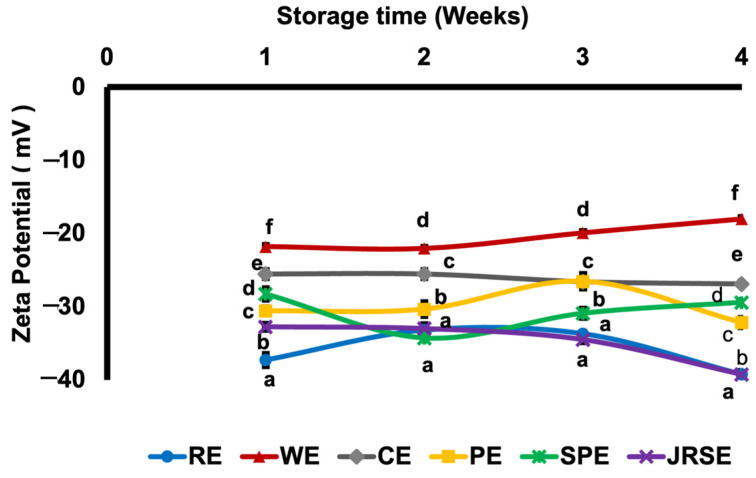
Zeta-potential of the GSD-stabilized soybean O/W emulsions during four weeks of storage at 25 °C.

**Figure 8:**
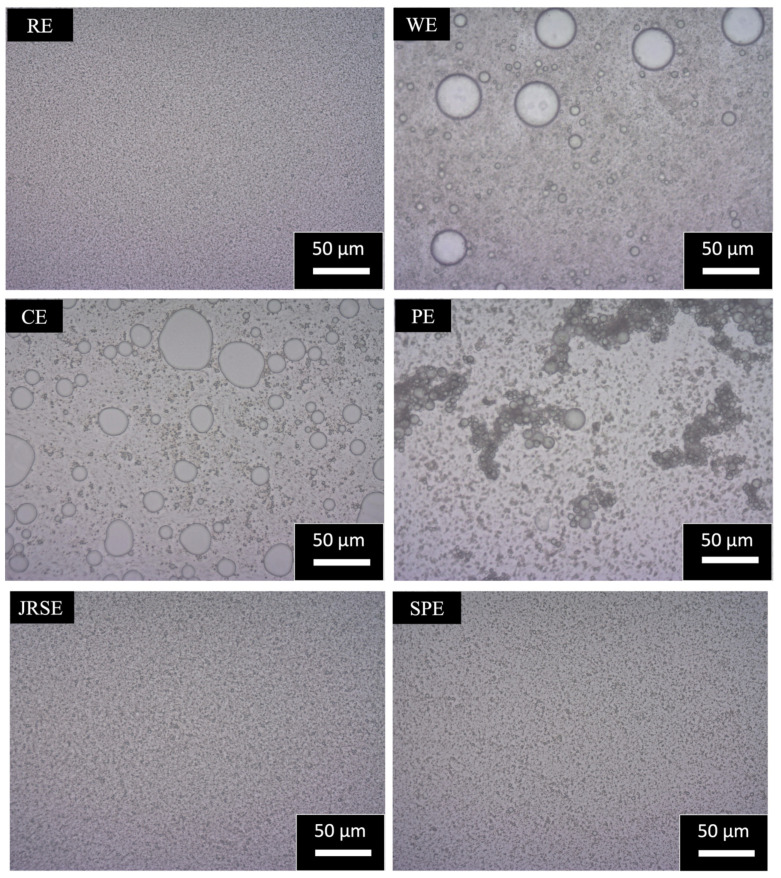
Optical micrographs of GSD-stabilized O/W emulsions on formulation day at 25 °C.

At 25 °C storage for four weeks, both RE and JRSE emulsions, with a d_4,3_ of approximately 1 μm (Figure 5), exhibited the highest stability. The viscosity increase fell within a suitable range, preventing the movement of oil droplets and their coalescence (Figure 6). Additionally, the ζ-potential (Figure 7) remained consistent at around −39.2 and −34.9 ± 0.45 mV, suggesting the maintenance of weak steric repulsion, which plays a crucial role in stability during storage, and the emulsions showed minimal creaming (Figure S10a,b). The increase in viscosity is due to the gelling and pasting properties of starch polysaccharides, breakdown of crystalline structures, and leaching of amylose, which are largely dependent on the molecular weight of starch. This observation was consistent with the findings of prior research [17]. CE was observed to be the third most stable (Figure S10b), with a d_4,3_ of approximately 10 μm. Similar to the above cases, the viscosity increase was within a suitable range. Additionally, it exhibited minimal creaming. However, the ζ-potential remained within a narrow range (−26.9 ± 0.26 mV) throughout the storage period. The least stable emulsions were the WE, SPE, and PE. Viscosity increased for two weeks and then decreased for WE and PE, possibly owing to the initiation of retrogradation because of their largest d_4,3_ among all the GSDs, whereas for SPE, no change in viscosity was observed, and its viscosity was the lowest among all the emulsions, possibly owing to certain physicochemical properties arising from the small d_4,3_ of SPGSD and its higher amylopectin content. The creaming phenomenon is due to the upward movement of dispersed phase droplets in emulsions during storage, and a cream layer is observed which indicates instability. No creaming was observed for RE, JRSE, and CE. Creaming indexes were observed to be 17.88 ± 0.71% for WE, 18.46 ± 0.88% for SPE, and 55.9 ± 1.75% for PE (Figure S10b), possibly due to their larger particle sizes (WE; d_4,3_: ~26 μm, PE; d_4,3_: ~14 μm), which can be attributed to the larger d_4,3_ of their respective GSDs used in emulsification. Although the d_4,3_ of SPE was rather small (~2 μm), it is likely that the higher amylopectin content in potato and sweet potato accelerated the retrogradation rate in the emulsions formulated with PGSD and SPGSD, resulting in creaming [21]. These three emulsions exhibited the lowest ζ-potential. For WE, ζ-potential reduced during storage, whereas for PE and SPE, ζ- potential (−29.4 *±* 0.62 mV and −32.2 *±* 0.25 mV, respectively) was maintained during the storage period. Optical micrographs (Figure 8) for all the freshly prepared emulsions show that dispersed phase droplets are well coated with GSDs and emulsion droplets are uniformly distributed. Creaming and oiling off were observed in the PE after four weeks of storage at 25 °C. A possible reason for this is the larger d_4,3_ of PE and the polysaccharide content of the corresponding GSD.

After four weeks of storage at 45 °C, all emulsions were found to be very unstable. ζ-potential (Figure S11) decreased during storage in all emulsions. Viscosity (Figure S12) increased exponentially for all emulsions and then decreased, possibly due to the disruption of amorphous and crystalline intermolecular alignments of the emulsion phases and initiation of the retrogradation process. An exponential increase in d_4,3_ (Figure S13) and noticeable creaming were observed in all emulsions (Figure S13a). Oiling off was also observed in PE. The stability of the emulsion may have been influenced not only by the retrogradation of the gelatinized starch fractions but also by the thermal vibrations of both the gelatinized starch fractions and the oil droplets. Typically, the retrogradation of gelatinized starch occurs when it is stored at less than 60 °C [23]. If emulsion stability were primarily influenced by starch retrogradation, one would expect all emulsions stored at various temperatures to exhibit instability. However, the only destabilized emulsion was the one stored at 45 °C. Furthermore, as the temperature increased, the size of the oil droplets also increased. Therefore, it appears more plausible that emulsion stability was affected primarily by thermal vibration. Optical micrographs (Figure S14b) show coalescence of dispersed droplets, cloudiness, and irregular structures, suggesting the rupture of intermolecular bonds due to thermal vibration.

### 2.4. Effect of Oil Weight Fraction on the Formulation and Stability of Indica Rice GSD Stabilized O/W Emulsions

**Figure 9:**
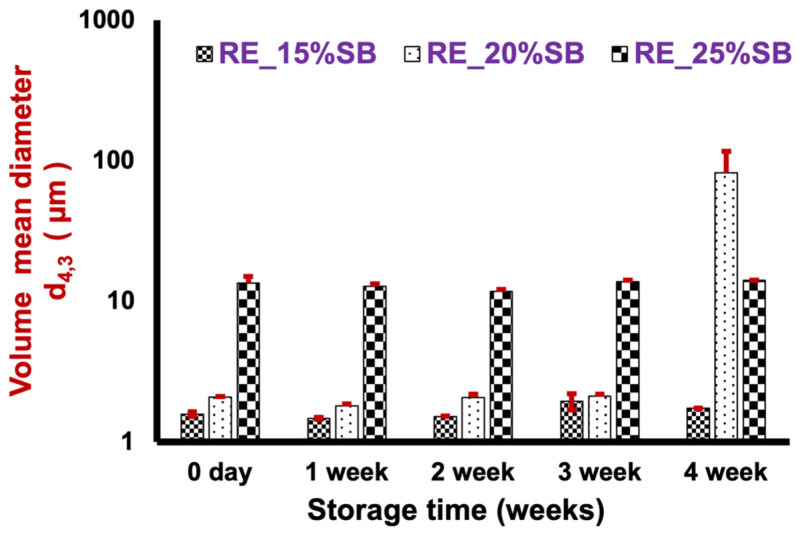
Volume mean diameter (d_4,3_) of the GSD-stabilized (high oil weight fraction) soybean O/W emulsions during four weeks of storage at 25 °C.

To investigate the emulsifying capability of RGSD, emulsions with higher weight fractions of soybean oil (15%, 20%, and 25% *w*/*w*) were formulated. RGSD was selected out of the six starches since the emulsions formulated with it exhibited the smallest particle size, fell within the optimum viscosity range, maintained a consistent surface ζ-potential, and showed the least creaming during storage at different temperatures. Emulsions containing 15% (*w*/*w*) soybean oil remained stable with a d_4,3_ of 1.57 μm (Figure 9), and least creaming was observed after four weeks of storage. In contrast, in 25% oil weight fraction emulsion, an increased d_4,3_ of 14.14 μm and creaming and oiling-off were observed (Figure S15). These findings align with those of a previous study [6], wherein a linear decrease in the stability of oil-in-water (O/W) emulsions was observed with an increase in both the volume of the oil phase and d_4,3_ function. The increase in the d_4,3_ value can be attributed to starch retrogradation in the continuous phase. In addition, the GSD particles in the continuous phase were not sufficient to coat the increasing concentration of the oil phase; therefore, the free oil droplets coalesced to form larger droplets. As a result, the stability decreased with increasing oil weight fractions, as is evident from the optical micrographs (Figure S15). The viscosity of *indica rice* GSD stabilized 15% soybean oil-in-water emulsion (RE_SB15%) and *indica rice* GSD stabilized 20% soybean oil-in-water emulsion (RE_SB20%) increased for the first two weeks and then decreased in the subsequent two weeks during storage at 25 °C, whereas for *indica rice* GSD stabilized 25% soybean oil-in-water emulsion (RE_SB25%), viscosity increased exponentially in the first week and then decreased (Figure S16). ζ-potential showed a decreasing trend after the second week, possibly due to lesser concentrations of GSDs with increasing oil weight concentration in emulsions, thereby reducing the ionic groups of GSDs responsible for electrical conductivity (Figure S17).

## 3. Materials and Methods

### 3.1. Materials

Six different native starches were selected (*indica* rice, Sigma-Aldrich Co. LLC; *japonica* rice, Joetsu Starch Co., Ltd., Nagaoka, Japan; wheat, corn, potato, and sweet potato, Wako Pure Chemical Industries, Osaka, Japan). Soybean oil, sodium azide (NaN_3_, antimicrobial agent) and Sudan IV (C_24_H_20_N_4_O, staining of lipid) were obtained from Wako Pure Chemical Industries, Ltd., Osaka, Japan. FITC dye was obtained from Sigma-Aldrich Co., St. Louis, MO, USA. All materials utilized were of analytical grade and were employed without additional purification.

### 3.2. Preparation of GSDs

Starch suspensions were prepared by adding each of the native starch powders 3% (*w*/*w*) to Milli-Q water 97% (*w*/*w*). Sodium azide 0.02% (*w*/*w*) was added to each starch suspension as an antimicrobial agent. The starch suspensions were gelatinized in a hot water bath (90 ℃) for 20 min, along with continuous stirring at 500 rpm using a propeller stirrer (IKA MINISTAR 20 Control, Osaka, Japan). Gelatinization temperature ranged from 85 to 90 ℃. Subsequently, the GSDs were cooled to room temperature and subjected to homogenization at 10,000 rpm for 5 min using a rotor-stator homogenizer (Polytron PT 10-35 GT, Kinematica AG, Lucerne, Switzerland).

### 3.3. Formulation of O/W Emulsions

The continuous phase, fresh GSD 90% (*w*/*w*), was mixed with the dispersed phase, 10% (*w*/*w*) soybean oil stained with 0.002% (*w*/*w*) of Sudan dye (for staining lipids). The soybean oil turned completely red after being stirred with a magnetic stirrer for 1 d. O/W emulsions were formulated using a rotor-stator homogenizer at 10,000 rpm for 5 min for primary emulsification. The resultant coarse emulsion was then immediately high-pressure homogenized (Microfluidizer, Microfluidics, Model M-110EH, Newton, MA, United States) at 100 MPa for 4–5 cycles for secondary emulsification. High dispersed phase O/W emulsions were also formulated as previously described, with soybean oil concentrations of 15%, 20%, and 25% (*w*/*w*) using RGSD as the continuous phase.

### 3.4. Determination of Average Particle Size and Particle-Size Distribution

Starch powders, GSDs, O/W emulsions, and highly dispersed-phase O/W emulsion droplets were examined using a laser light-scattering size analyzer (Beckman Coulter LS 13 320, Miami, FL, USA), and the mean droplet size was quantified in terms of the *d*_4,3_, defined as follows:

$$d_{4,3}=\sum n_{i}{d_{i}}^{4}/\sum n_{i}{d_{i}}^{3}$$

where $d_{i}$ represents the diameter of the *i*-th measured droplet and n denotes the number of measured droplets (*n* = 100). The droplet size distribution of the O/W emulsion was represented by the relative span factor (*RSF*), defined as follows:

$$RSF=(d_{90}-d_{10})/d_{50}$$

The particle size distribution was measured four times for each condition.

### 3.5. Morphological, Compositional, and Thermal Properties Analysis of Starch Powders

The morphologies of all starch powders were observed using a scanning electron microscope (Hitachi tabletop microscope TM-1000; Ibaraki, Japan). Micrographs were captured at various scales for each sample. We selected the clearest and most representative micrographs at the same magnification of 1200× for each sample. The polysaccharide composition of each starch powder was determined using the Megazyme Con A method [20,24]. The starch was pretreated for lipid removal. Then, the Con A precipitate of amylopectin and amylose was enzymatically hydrolyzed into D-glucose. The resulting D-glucose was analyzed using a glucose oxidase/peroxidase reagent. Similarly, in a separate aliquot of the acetate/salt solution, total starch was hydrolyzed to D-glucose and measured colorimetrically using the same glucose oxidase/peroxidase reagent. Amylose concentration in the starch sample was estimated by comparing the absorbance of the supernatant from the Con A-precipitated sample at 510 nm with that of the total starch sample.

The thermal properties of all the starch powders were assessed using a differential scanning calorimeter (DSC 8500, PerkinElmer Co., Ltd., Norwalk, CT, USA). Approximately 10–15 mg of each starch sample was placed in an aluminum capsule, and 45 μL Milli-Q water was added to reach a sample concentration of 25% (*w*/*w*). The aluminum capsule was sealed, and measurements were made between 40 °C and 140 °C at a heating rate of 10 °C min^−1^.

### 3.6. Interfacial Tension Measurement

Before conducting measurements for interfacial tension, the densities of the GSDs at a concentration of 3% (*w*/*w*) and soybean oil were determined using a density/specific gravity meter (DA-130N, Kyoto Electronics Co., Ltd., Kyoto, Japan). Subsequently, the interfacial tension between all GSDs at 3% (*w*/*w*) concentration and soybean oil was assessed using the pendant drop method (DM-501, Kyowa Interface Science Co., Ltd., Saitama, Japan) at room temperature. GSD samples were introduced into a glass syringe and dispensed to create a pendant drop at the end of a stainless-steel needle (22G) submerged in the oil phase. The interfacial tension was then automatically calculated using FAMAS analysis software version 5.1.1, considering the density disparity between the two phases, droplet shape, and size immediately after reaching the maximum volume and before detachment, as determined by the Young-Laplace equation.

### 3.7. Viscosity Measurement of GSDs and O/W Emulsion

Viscosities of the GSDs and O/W emulsions were determined using a viscometer (Brookfield DV-II+ Pro; Middleboro, MA, USA). A volume of 6.7 mL sample was placed into the chamber, and the spindle (SC4-18, diameter: 17.5 mm, height: 31.7 mm) was rotated at 0.5–40 rpm. The viscosity value was obtained and recorded 30 s after spindle rotation. As emulsion viscosity is sensitive to temperature, a water circulating thermostatic bath was used to maintain the emulsion temperature at 5 °C, 25 °C, and 45 °C.

### 3.8. Surface Zeta-Potential Measurement of Emulsions

The ζ-potential of all emulsions was analyzed using a Zetasizer Nano ZS electrophoresis instrument (Malvern Instruments Ltd., Worcestershire, UK). After allowing the emulsions to settle for 24 h, the samples were diluted with ultrapure water (at a ratio of 1:100) to minimize multiple scattering effects. Following dilution, the samples were loaded into a folded 1 mL capillary cell. The refractive indices of the aqueous and dispersed phases were 1.330 and 1.432, respectively.

### 3.9. Morphological Analysis

The morphologies of GSDs, oil-in-water (O/W) emulsions, and highly dispersed O/W emulsions were examined using an optical microscope (DFC300FX; Leica Microsystems, Wetzler, Germany). This analysis focused on observing the microstructure, droplet distribution, starch retrogradation, and emulsion coalescence, which were the key parameters investigated in this study.

### 3.10. Fluorescent Microscopy

The microstructures of RGSD stabilized O/W emulsion droplets formulated under optimized conditions were observed using a fluorescence microscope (KEYENCE, BZ-8000, Tokyo, Japan). Before homogenization, RGSD was stained with a small amount of 0.002% FITC dye overnight at ambient temperature. All fluorescence images were captured using a 20× objective (numeric aperture 1.30).

### 3.11. Stability Test

Storage stability was evaluated at 25 °C for 4 weeks for the formulated GSDs, O/W emulsions, and high dispersed phase O/W emulsions. In addition, the storage stability of O/W emulsions at different temperatures (5 °C and 45 °C) for 4 weeks was also evaluated. The temperature conditions mentioned above were selected to evaluate emulsion stability at room temperature, cold temperature, and at elevated temperature. The chosen time period of 4 weeks was considered to be a balanced timeframe for stability assessment, aiming to avoid practical constraints. The emulsions under storage were evaluated weekly for *d*_4,3_ and particle size distribution, viscosity, ζ-potential, morphological changes, and visual appearance. The creaming index of O/W emulsions was determined by storing the emulsions in screw-capped bottles for 9 days at 25 °C [6]. The creaming index was calculated using the following equation:

$$\mathrm{Creaming}\;\mathrm{Index}\;(\%)=\frac{H_{S}}{H_{E}}\times 100$$

Here, *H_s_* (mm) is the height of the serum layer, and *H_E_* (mm) is the height of the emulsions.

### 3.12. Statistical Analysis

The experiments were conducted in triplicate, and the results were expressed as the mean and standard deviation of the measurements. Statistical analysis was performed using one-way analysis of variance (ANOVA) with Statistix 8.1 software (Tallahassee, FL, USA), and the least significant difference (LSD) was determined at a confidence level of 95%. Different letters indicate significant differences (*p* < 0.05).

## 4. Conclusions

According to the findings of this study, starch employed as a gelatinized starch dispersion can be utilized as-is as a natural emulsifying material for O/W emulsions without a synthetic emulsifier or other natural cosurfactants. We confirmed that *indica* rice, *japonica* rice, and corn GSDs were capable of producing submicron and micron emulsions with reduced interfacial tension, consistent negative ζ-potential, optimum viscosity range, and good physical stability under one month of storage at room temperature and low temperature storage. We also investigated the emulsification ability of *indica* rice GSD with higher oil-weight fractions. Emulsification with 15% *w*/*w* soybean oil remained stable at micron level *d*_4,3_, and minimal creaming was observed even after four weeks of storage at room temperature. Gelatinization, as a physical modification of starch, is a suitable technique for stabilizing emulsions and meeting clean-label standards for product optimization.

Future research could broaden the scope of this study by examining the oxidative stability, encapsulation efficiency, and digestive efficacy of GSD-stabilized emulsions. This would enable the exploration of diverse potential applications for emulsions stabilized using natural ingredients, such as the food, pharmaceutical, and cosmetic industries, as well as in other sectors aspiring for high-quality, high-value, and naturally processed products in a cost-effective manner.

## Data Availability

The data presented in this study are available on request from the corresponding author.

## Supplementary Materials

The following supporting information can be downloaded at: https://www.mdpi.com/article/10.3390/molecules29091923/s1, Figure S1. Variation in volume mean diameter (d_4,3_) of starch powder and gelatinized starch dispersions (GSDs); Figure S2. Optical micrographs of GSDs at 25 °C on formulation day. RGSD, *indica* rice gelatinized starch dispersion; WGSD, wheat gelatinized starch dispersion; CGSD, corn gelatinized starch dispersion; SPGSD, sweet potato gelatinized starch dispersion; PGSD, potato gelatinized starch dispersion; JRSGSD, *japonica* rice gelatinized starch dispersion; Figure S3. Viscosity at formulation day (var. with shear rates for RGSD, WGSD, CGSD, SPGSD, PGSD, and JRSGSD); Figure S4. Scanning electron microscope (SEM) images of the starch powders. RS, *indica* rice starch; JRS, *japonica* rice starch; WS, wheat starch; CS, corn starch; PS, potato starch; SPS, sweet potato starch; Figure S5. Formulation day viscosity of the GSD-stabilized soybean oil-in-water (O/W) emulsion. CE: corn GSD-stabilized (O/W) emulsion, SPE: Sweet potato GSD-stabilized (O/W) emulsion, PE: potato GSD-stabilized (O/W) emulsion, JRSE: *japonica* rice GSD-stabilized (O/W) emulsion, RE: *indica* rice GSD stabilized (O/W) emulsion, WE: Wheat GSD stabilized (O/W) emulsion; Figure S6. Zeta (ζ–) potential of the GSD-stabilized soybean oil-in-water (O/W) emulsions during four weeks of storage at 5 °C; Figure S7. Viscosity of the GSD-stabilized soybean oil-in-water (O/W) emulsions during four weeks of storage at 5 °C; Figure S8. Volume mean diameter (d_4,3_) of the GSD-stabilized soybean oil-in-water (O/W) emulsions during four weeks of storage at 5 °C; Figure S9. Optical micrographs of GSD stabilized O/W emulsions after four weeks of storage at 5 °C (RE: *indica* rice GSD stabilized (O/W) emulsion, WE: Wheat GSD stabilized (O/W) emulsion, CE: Corn GSD stabilized (O/W) emulsion, SPE: Sweet potato GSD stabilized (O/W) emulsion, PE: Potato GSD stabilized (O/W) emulsion, JRSE: *japonica* rice GSD stabilized (O/W) emulsion. Figure S10. Images of GSD-stabilized O/W emulsions on formulation day (a) and after four weeks of storage at 25 °C (b); Figure S11. Zeta (ζ –) potential of the GSD-stabilized soybean oil-in-water (O/W) emulsions during four weeks of storage at 45 °C; Figure S12. Viscosity of the GSD-stabilized soybean oil-in-water (O/W) emulsions during four weeks of storage at 45 °C; Figure S13.Volume mean diameter (d_4,3_) of the GSD-stabilized soybean oil-in-water (O/W) emulsions during four weeks of storage at 45 °C; Figure S14. Images (a) and optical micrographs (b) of GSD stabilized O/W emulsions after four weeks of storage at 45 °C; Figure S15. Images and optical micrographs of the GSD-stabilized (high oil weight fraction) emulsions after four weeks of storage at 25 °C. RE_15%, RE_20%, and RE_25% indicate *indica* rice GSD-stabilized (O/W) emulsions at 15%, 20%, and 25% soybean oil weight fractions, respectively; Figure S16. Viscosity of the GSD-stabilized (high oil weight fraction) soybean oil-in-water (O/W) emulsions during four weeks of storage at 25 °C; Figure S17. Zeta-potential of the GSD-stabilized (high oil weight fraction) soybean oil-in-water (O/W) emulsions during four weeks of storage at 25 °C; Table S1. Polysaccharide composition of starch (Megazyme Con A method); Table S2. Zeta potential of the GSD-stabilized O/W emulsion on the day of formulation; Table S3. Thermal properties of native starch powders.
