# Supplementary material for: Formulation and Characterization of Soybean Oil-in-Water Emulsions Stabilized Using Gelatinized Starch Dispersions from Plant Sources
Source: Molecules. 2024 Apr 23;29(9):1923. doi: 10.3390/molecules29091923 (PMC11085249; doi:10.3390/molecules29091923)
Supplement: Supplementary file 1 [file molecules-29-01923-s001.zip › molecules-2963950-supplementary.pdf]

Supplementary Information

Formulation, Characterization, and Storage Evaluation of Oil-in-Water Emulsions Stabilized Using Gelatinized Starch Dispersions from Plant Sources

Ankita Singh [1,2], Takumi Umeda [1] and Isao Kobayashi [1,3,*]

[1] Institute of Food Research, National Agriculture and Food Research Organization, 2-1-12 Kannodai, Tsukuba 305-8642, Ibaraki, Japan
[2] Graduate School of Science and Technology, University of Tsukuba, 1-1-1 Tennodai, Tsukuba 305-8572, Ibaraki, Japan
[3] School of Integrative and Global Majors, University of Tsukuba, 1-1-1 Tennodai, Tsukuba 305-8577, Ibaraki, Japan

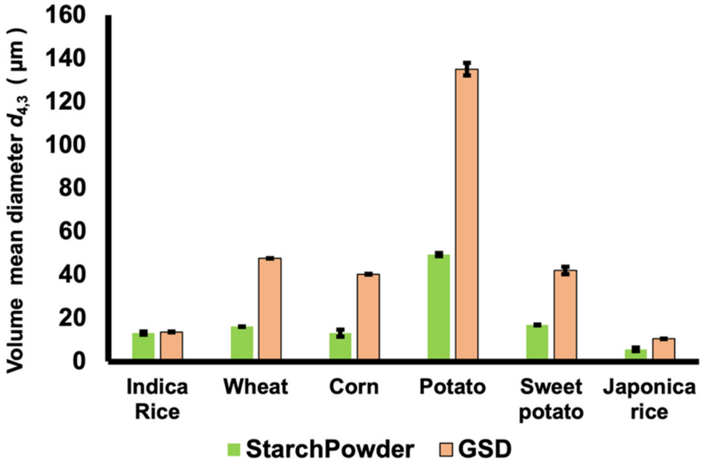

Figure S1. Variation in volume mean diameter ($d_{4,3}$) of starch powder and gelatinized starch dispersions (GSDs)

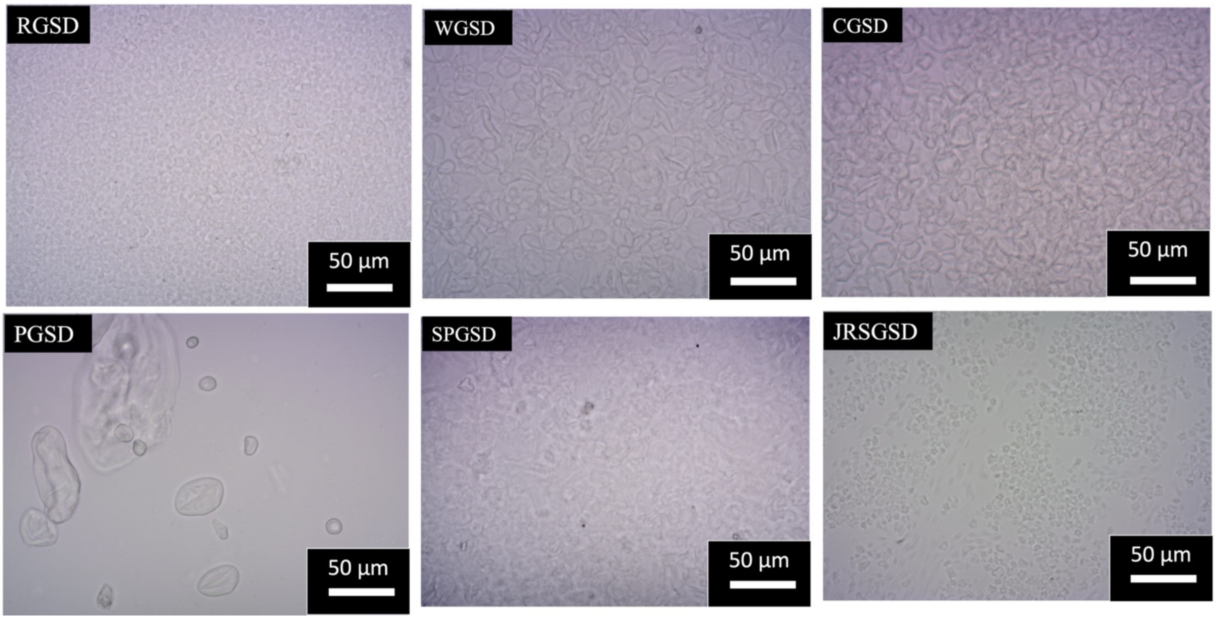

Figure S2. Optical micrographs of GSDs at 25°C on formulation day. RGSD, *indica* rice gelatinized starch dispersion; WGSD, wheat gelatinized starch dispersion; CGSD, corn gelatinized starch dispersion; SPGSD, sweet potato gelatinized starch dispersion; PGSD, potato gelatinized starch dispersion; JRSGSD, Japonica rice gelatinized starch dispersion

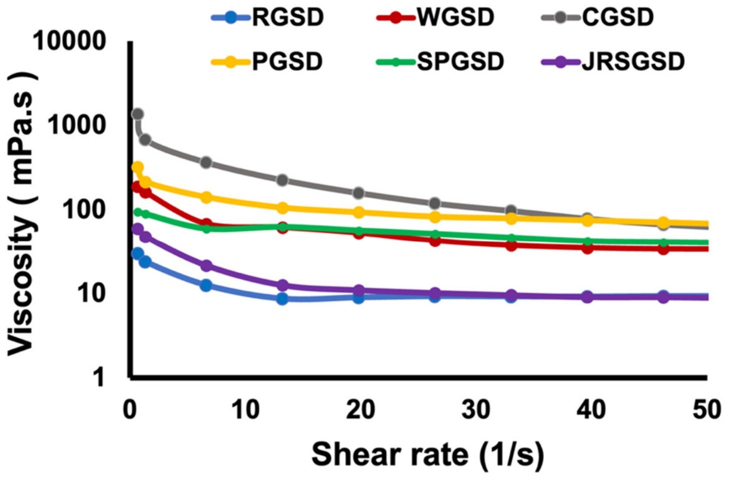

Figure S3. Viscosity at formulation day (var. with shear rates for RGSD, WGSD, CGSD, SPGSD, PGSD, and JRSGSD)

Table S1. Polysaccharide composition of starch (Megazyme Con A method)

Polysaccharides	*Indica* rice	Wheat	Corn	Potato	Sweet potato	*Japonica* rice
Amylose (% (w/w))	20.61± 0.48	26.25± 1.25	29.85±1.55	14.80±0.10	19.9±0.20	13.85±0.55
Amylopectin (% (w/w)	79.28±0.58	73.7±1.2	70.1±1.6	85.15±0.05	80.05±0.55	86.15±0.55

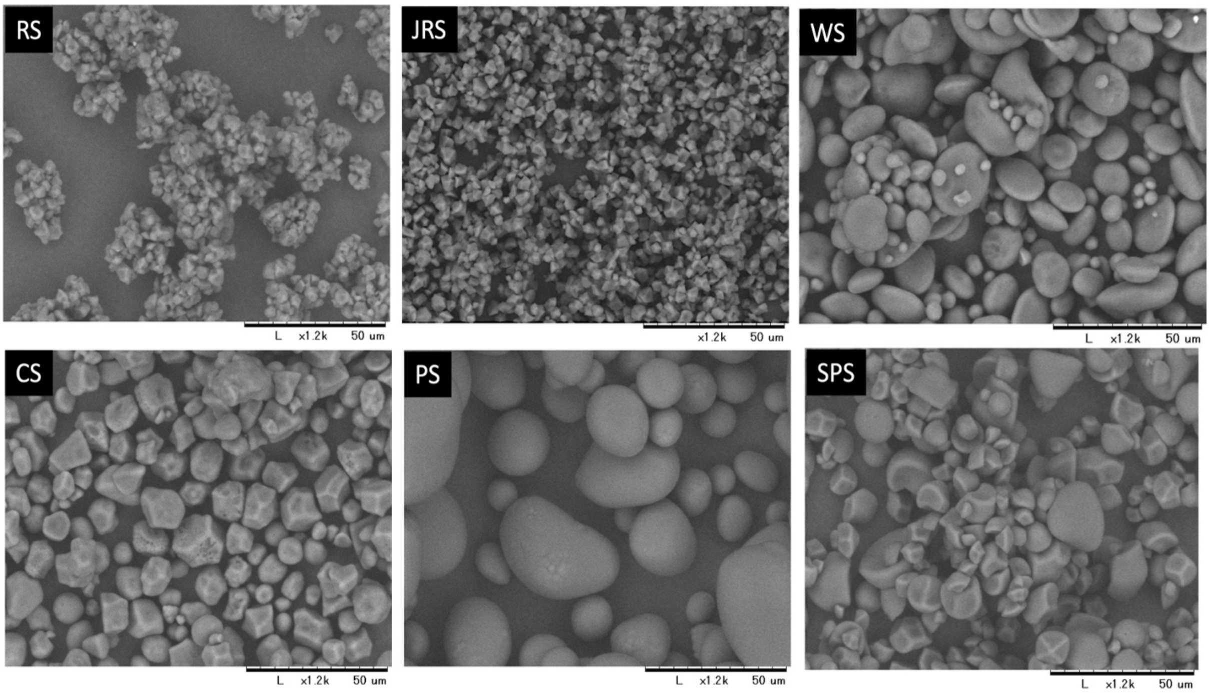

Figure S4. Scanning electron microscope (SEM) images of the starch powders. RS, indica rice starch; JRS, japonica rice starch; WS, wheat starch; CS, corn starch; potato starch; SPS, sweet potato starch;

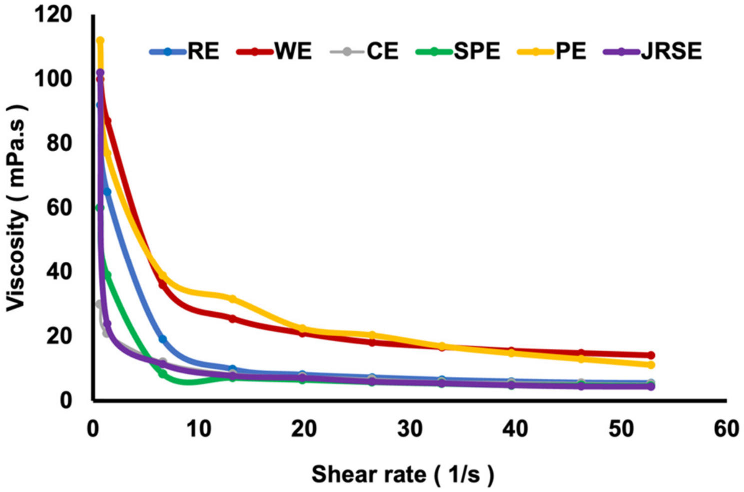

Figure S5. Formulation day viscosity of the GSD-stabilized soybean oil in water (O/W) emulsion. CE: corn GSD-stabilized (O/W) emulsion, SPE: Sweet potato GSD-stabilized (O/W) emulsion, PE: potato GSD-stabilized (O/W) emulsion, JRSE: *japonica* rice GSD-stabilized (O/W) emulsion, RE: *indica* rice GSD stabilized (O/W) emulsion, WE: Wheat GSD stabilized (O/W) emulsion

Table S2. Zeta potential of the GSD-stabilized O/W emulsion on the day of formulation

GSD stabilized O/W emulsion	Zeta-potential (mV)
RE	-32.4±1.72
WE	-28±1.58
CE	-27.8±1.66
PE	-32.3±0.46
SPE	-29.4±0.29
JRSE	-41.2±0.78

Table S3. Thermal properties of native starch powders

**Starch**	**T_o (°C)**	**T_p (°C)**	**T_c (°C)**	**ΔH_{gel} (J g^{-1})**
Rice (*Indica*)	59.765±0.33	68.7±0.22	79.66±0.6	11.87±0.67
Rice (*Japonica*)	63.59±0.42	71.72±0.41	79.67±0.29	14.43±0.85
Wheat	56.04±0.02	63.35±0.57	71.21±0.53	10.74±0.05
Corn	66.91±0.41	72.38±1.03	80.07±1.03	13.51±0.25
Potato	62.71±0.27	68.71±0.71	76.48±0.93	18.52±1.89
Sweet Potato	67.83±0.39	79.27±0.23	87±0.07	15.41±0.46

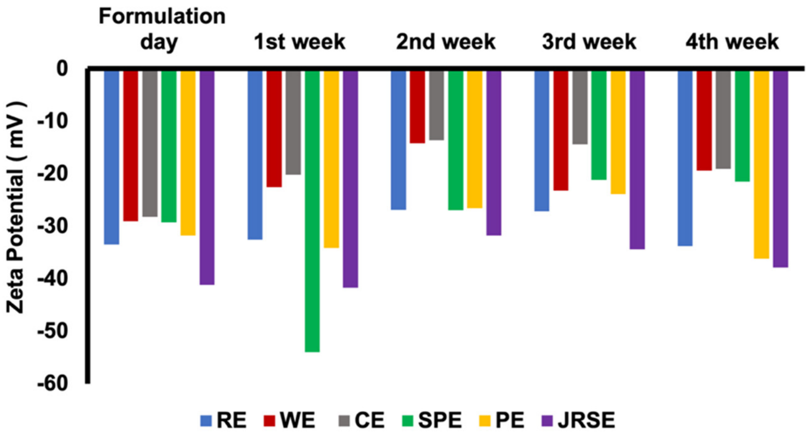

Figure S6. Zeta (ζ –) potential of the GSD-stabilized soybean oil in water (O/W) emulsions during 4 weeks of storage at 5 °C

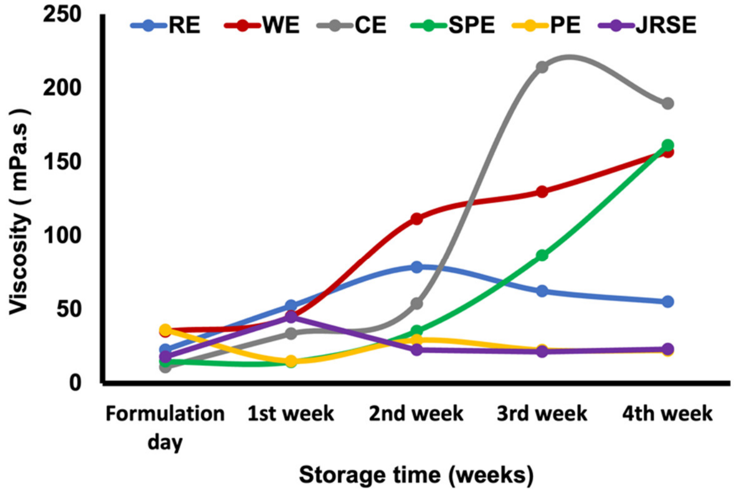

Figure S7. Viscosity of the GSD-stabilized soybean oil in water (O/W) emulsions during 4 weeks of storage at 5 °C

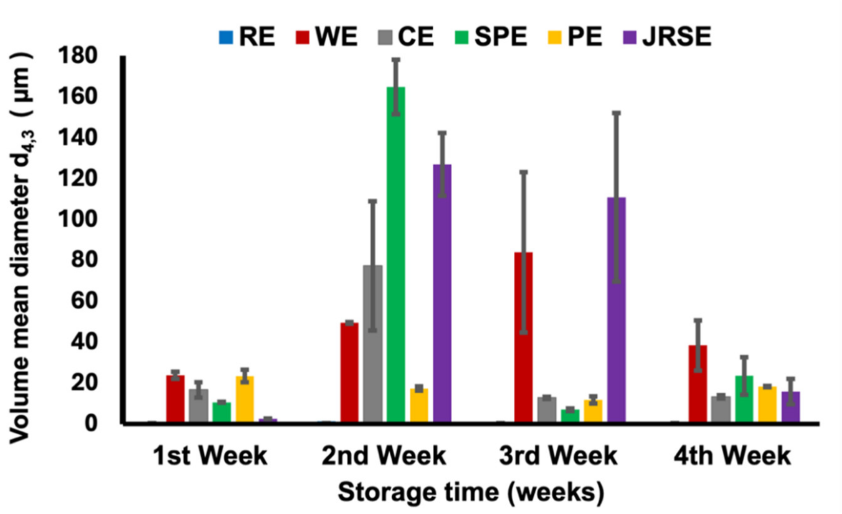

Figure S8. Volume mean diameter ($d_{4,3}$) of the GSD-stabilized soybean oil in water (O/W) emulsions during 4 weeks of storage at 5 °C

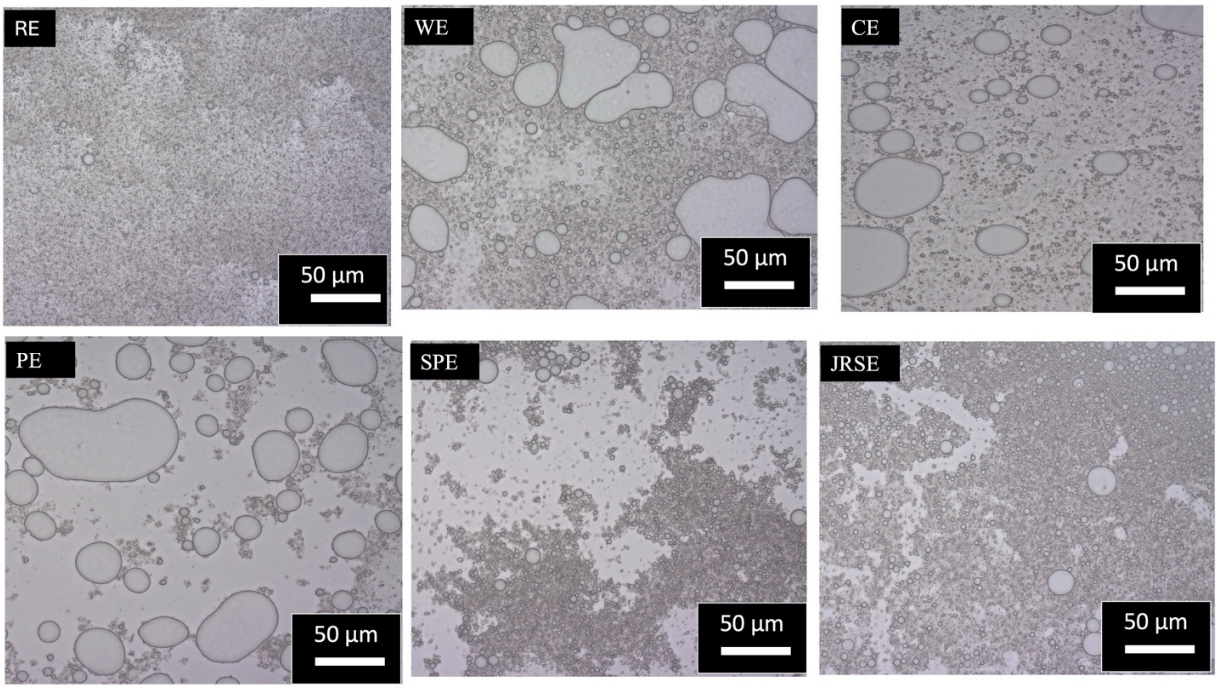

Figure S9. Optical micrographs of GSD stabilized O/W emulsions after 4 weeks of storage at 5°C (RE: *Indica* rice GSD stabilized (O/W) emulsion, WE: Wheat GSD stabilized (O/W) emulsion, CE: Corn GSD stabilized (O/W) emulsion, SPE: Sweet potato GSD stabilized (O/W) emulsion, PE: Potato GSD stabilized (O/W) emulsion, JRSE: *Japonica* rice GSD stabilized (O/W) emulsion.

(a)

(b)

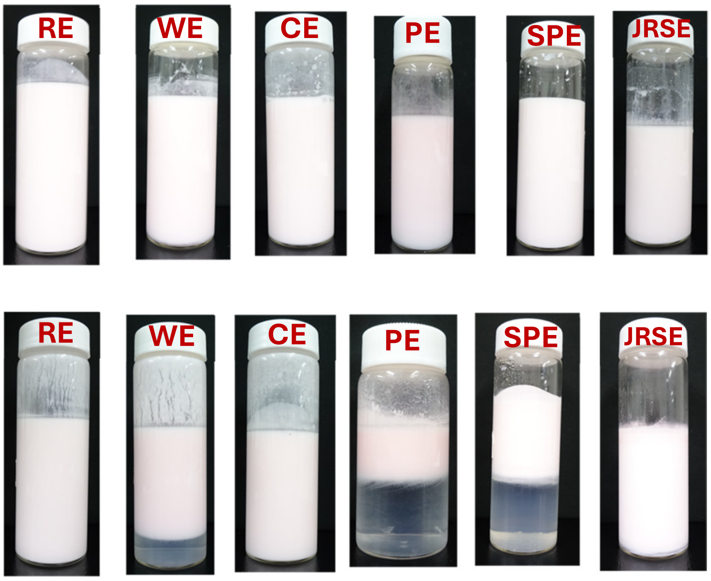

Figure S10. Images of GSD-stabilized O/W emulsions on formulation day (a) and after 4 weeks of storage at 25°C (b)

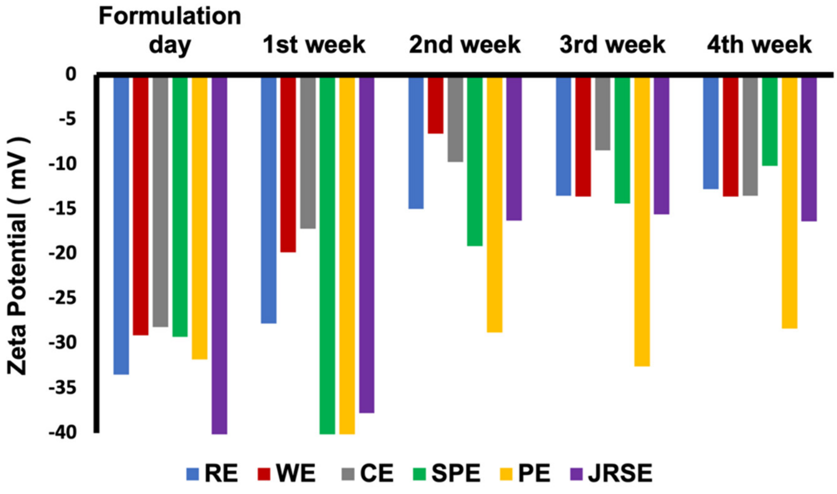

Figure S11. Zeta (ζ –) potential of the GSD-stabilized soybean oil in water (O/W) emulsions during 4 weeks of storage at 45 °C

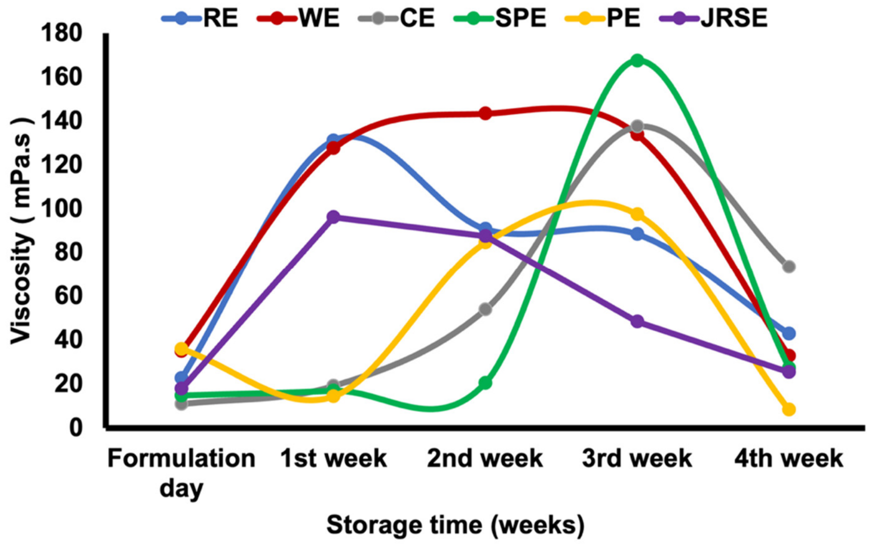

Figure S12. Viscosity of the GSD-stabilized soybean oil in water (O/W) emulsions during 4 weeks of storage at 45 °C

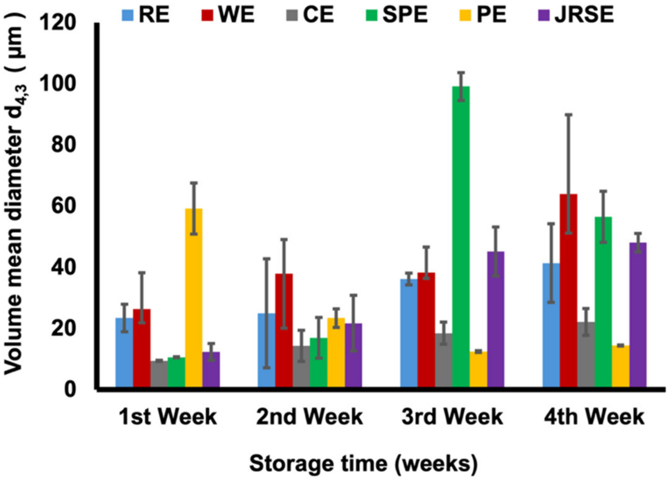

Figure S13. Volume mean diameter ($d_{4,3}$) of the GSD-stabilized soybean oil in water (O/W) emulsions during 4 weeks of storage at 45 °C

(a)

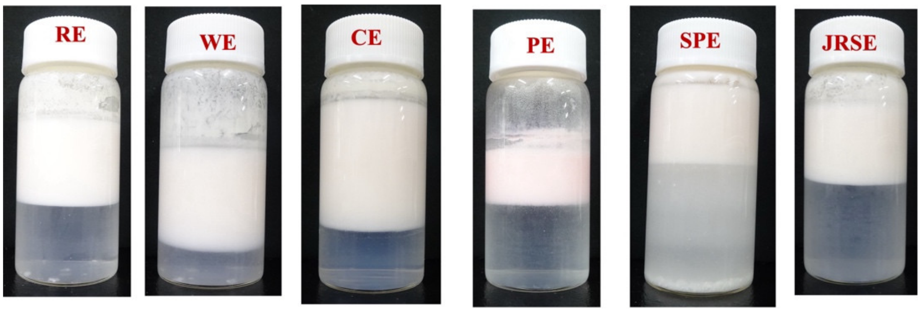

(b)

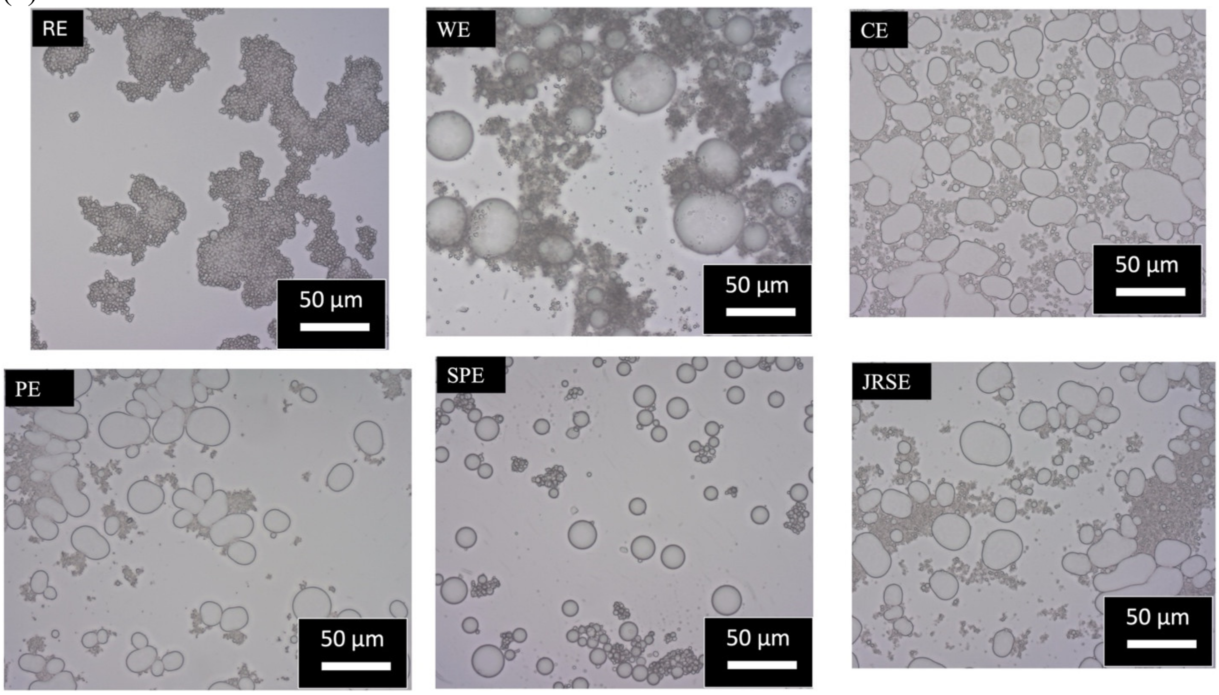

Figure S14. Images (a) and optical micrographs (b) of GSD stabilized O/W emulsions after 4 weeks of storage at 45°C

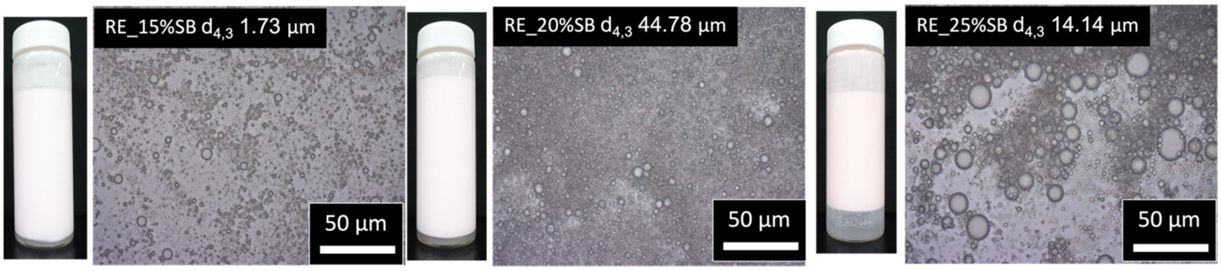

Figure S15. Images and optical micrographs of the GSD-stabilized (high oil weight fraction) emulsions after 4 weeks of storage at 25 °C. RE_15%, RE_20%, and RE_25% indicate *indica* rice GSD-stabilized (O/W) emulsions at 15%, 20%, and 25% soybean oil weight fractions, respectively

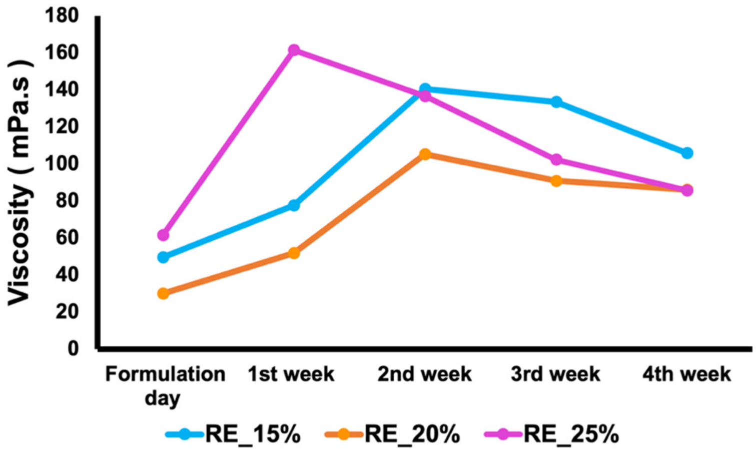

Figure S16. Viscosity of the GSD-stabilized (high oil weight fraction) soybean oil in water (O/W) emulsions during 4 weeks of storage at 25 °C

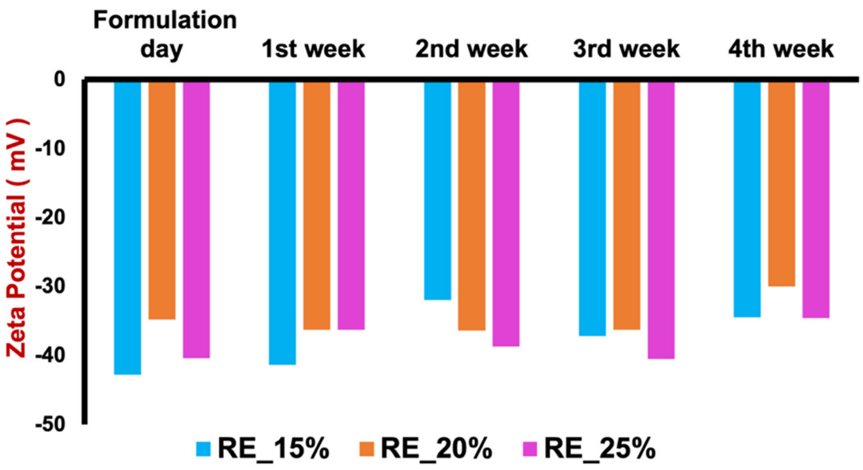

Figure S17. Zeta-potential of the GSD-stabilized (high oil weight fraction) soybean oil in water (O/W) emulsions during 4 weeks of storage at 25 °C
